# Imaging features of ALK-positive histiocytosis with neurological involvement: a case report and literature review

**DOI:** 10.3389/fonc.2024.1333519

**Published:** 2024-02-22

**Authors:** Juan Wang, Yan Zheng, Ying Xiong

**Affiliations:** ^1^ Department of Radiology, Tongji Hospital, Tongji Medical College, Huazhong University of Science and Technology, Wuhan, China; ^2^ Department of Pathology, The Fifth Hospital of Xiamen, Xiamen, China

**Keywords:** ALK-positive histiocytosis, nervous system, imaging features, magnetic resonance imaging, children

## Abstract

**Background:**

ALK-positive histiocytosis is an exceptionally rare neoplasm of histiocytes that predominantly involves the nervous system and can also affect the skin and other parts of the body. Previous relevant literature has provided limited information regarding the imaging manifestations of this disease with neurological involvement.

**Methods:**

We reported a case of ALK-positive histiocytosis with multisystem involvement. Together with a comprehensive literature review, the imaging characteristics of this disease in the nervous system were summarized.

**Results:**

A 3-year-old girl with abdominal pain and ambulation difficulty checked in at the Department of Pediatric Neurology. The initial diagnosis was “acute cerebellitis with ataxia” based on the elevated protein level in the cerebrospinal fluid (CSF). However, despite 3 months of treatment, her condition deteriorated. MRI showed an oval-shaped, intradural extramedullary nodule at the T6–T7 level. The patient was ultimately diagnosed as ALK-positive histiocytosis, accompanied by cauda equina and skin involvement. The literature review showed a total of 23 patients who had involvement of the nervous system and provided imaging descriptions. Together with our case, the imaging features were summarized as follows: iso-dense or slightly hyperdense on computed tomography (CT), isointense or iso-hypointense on T2-weighted imaging (T2WI), moderate homogeneous enhancement with mildly/markedly punctate enhancement or/and smooth ring enhancement on contrast-enhanced T1-weighted imaging (T1WI), restricted diffusion on diffuse weighted imaging (DWI), and elevated fluorodeoxyglucose (FDG) uptake on positron-emission tomography/computed tomography (PET/CT).

**Conclusion:**

The multimodal imaging findings of ALK-positive histiocytosis exhibit distinct characteristics, familiarity with which will enhance radiologists’ expertise and facilitate accurate diagnosis of this disease.

## 1 Introduction

ALK-positive histiocytosis (APH), a rare non-Langerhans histiocytosis, was initially reported in 2008 and classified as a tumor category of histiocyte/macrophage in the 5th edition of the WHO Classification of Tumors of the Lymphatic and Hematopoietic System in 2022 (1). The age at onset and site of involvement exhibited significant variability. However, more cases were seen in children, particularly female infants (2), with a greater prevalence in the nervous system. It could be categorized into a single-system and multisystemic disease based on the site of involvement. To date, less than 100 cases of APH have been reported. Previous relevant literature primarily focused on genetic characteristics, histopathology, treatment, and prognosis (2–6), with limited descriptions regarding imaging manifestations of this disease (7).

## 2 Materials and methods

### 2.1 Case description

**Figure 1:**
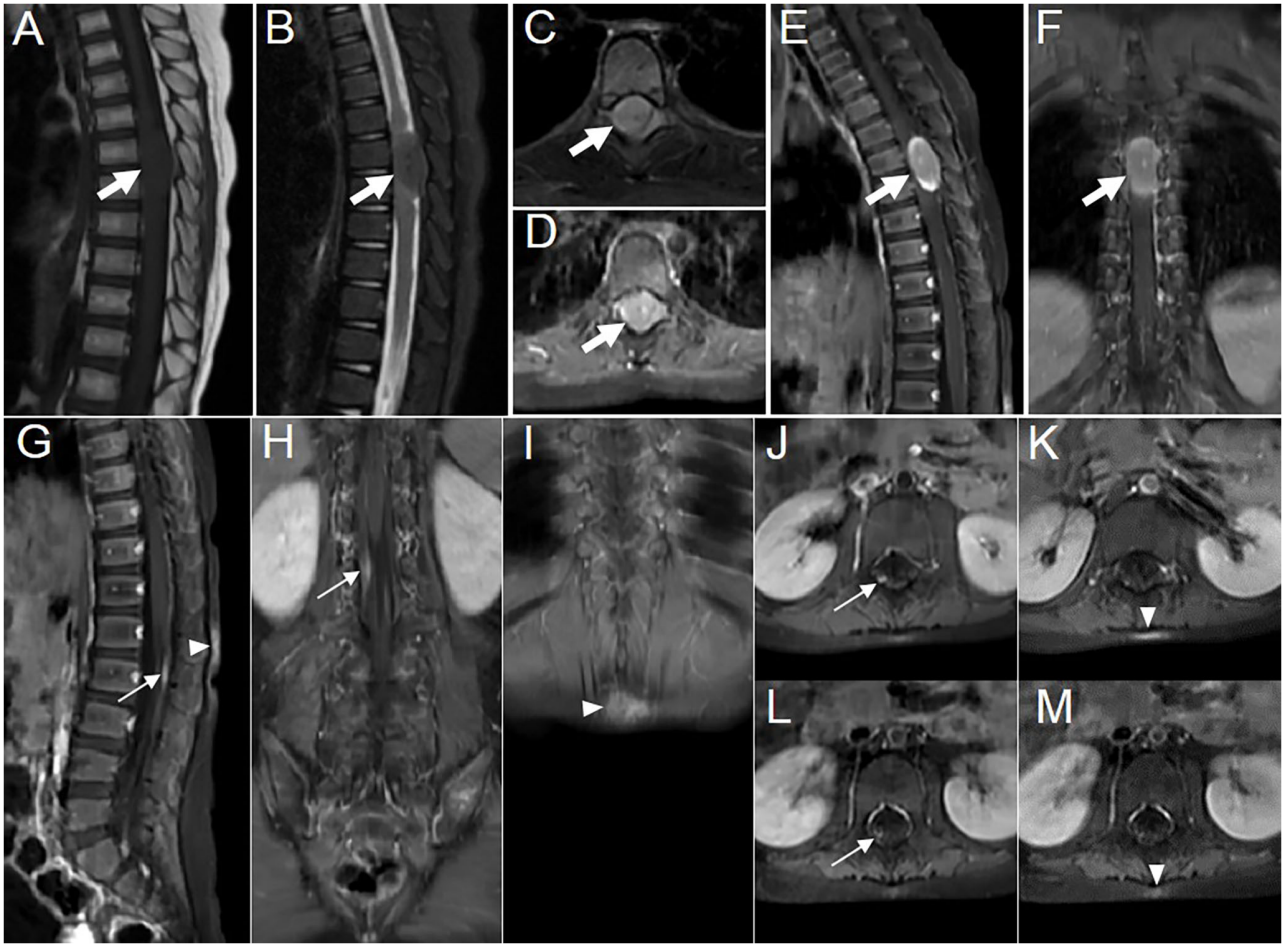
Images showing multisystem lesions in the 3-year-old girl with APH. **(A–C)** MR images showing hypointense on sagittal T1WI (**A**, thick arrow) and isointense with central punctate hypointense areas on sagittal and axial fat-suppressed sequences of T2WI (**B, C**, thick arrow). **(D–F)** Contrast-enhanced T1WI revealing moderate enhancement of the intradural extramedullary nodule, with marked enhancement at the upper and lower rims, accompanied by markedly punctate enhancement in the center on axial (**D**, thick arrow), sagittal (**E**, thick arrow), and coronal (**F**, thick arrow) scans. **(G–I)** Contrast-enhanced T1WI showing segmentally moderate enhancement of the lesions in the leptomeninges (thin arrow) and the dorsal skin at the L1–2 level (arrowhead) on sagittal **(G)** and coronal **(H, I)** scans. Axial contrast-enhanced T1WI showing the changes in leptomeninges and skin lesions before **(J, K)** and after **(L, M)** treatment, respectively.

A 3-year-old girl with abdominal pain lasting for over 2 weeks and ambulation difficulty for 3 days checked in at the Department of Pediatric Neurology. She had a history of novel coronavirus infection 1 month prior. Laboratory tests revealed a positive result in the qualitative analysis of CSF protein. CSF biochemistry showed total protein >6,000 mg/L ↑ and albumin 7,628 mg/L ↑. Complete set of CSF immunization results: IgG 537.0 mg/L ↑, IgM 58.1 mg/L ↑, IgA 19.4 mg/L ↑. Eight antibodies related to autoimmune cerebellar ataxia were tested negative in both serum and CSF samples. The patient was diagnosed as “acute cerebellitis with ataxia” and showed improvement after human immunoglobulin therapy and hormonal treatment. However, more than 3 months later, the patient presented leg weakness, walking difficulties, active tendon reflexes bilaterally, as well as positive Babinski’s sign. CSF routine examination revealed a qualitative presence of protein along with an increased number of nucleated cell (23 × 10^6^/L↑). CSF biochemistry showed decreased glucose level (2.64 mmol/L ↓) and elevated total protein (1,055 mg/L ↑) and albumin (697 mg/L ↑) level. The EBV antibody (IgG) and nucleic acid (EBV-DNA) tests of peripheral blood yielded positive results. A subsequent MRI examination revealed an oval-shaped nodule (approximately 27 mm × 16 mm × 13 mm) located in the intradural extramedullary space at the T6–T7 level, which was compressing the spinal cord (**Figures 1A–F**). Additionally, there was segmental enhancement of leptomeninges along the cauda equina and lumbar dorsal skin (**Figures 1G–K**).

**Figure 2:**
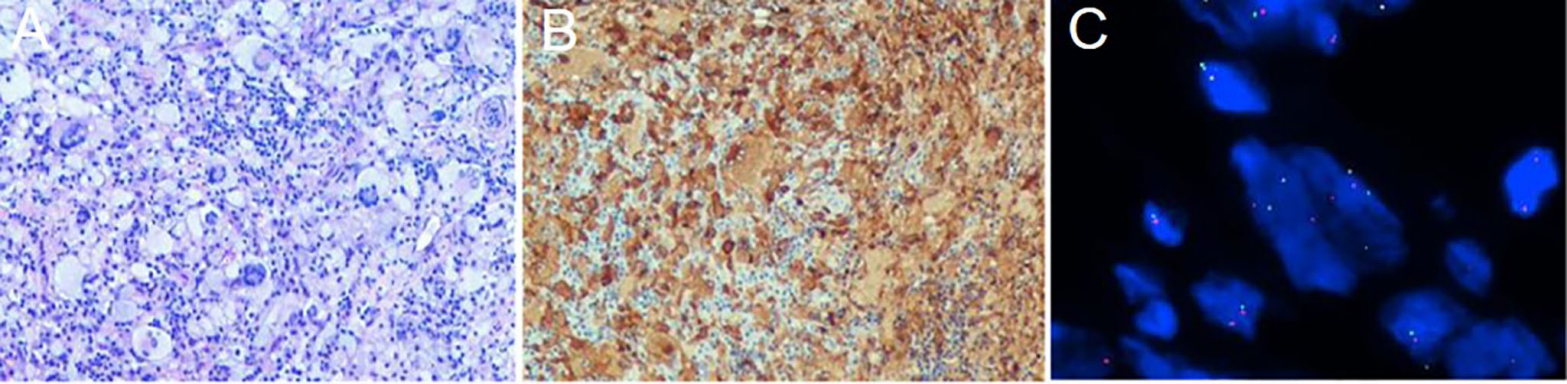
Histological and molecular findings from the biopsy of the intradural extramedullary nodule. (**A**, ×100) Hematoxylin and eosin staining showing hyperplasia of eosinophilic histiocytes, accompanied by the expression of ALKA4 (**B**, ×100). (**C**, ×1000) FISH (fluorescence *in situ* hybridization) analysis using ALK break-apart probe demonstrating the presence of ALK rearrangement with a positive rate of 28%.

The patient subsequently underwent an excision of the intradural extramedullary nodule. Intraoperatively, it was observed that the tumor appeared smooth, white, and soft with moderate blood supply. It adhered tightly to the dura mater and had a clear border from the spinal cord as it grew towards the intervertebral foramen. Finally, based on histopathological and molecular examination (**Figure 2**), the case was diagnosed as APH. Immunohistochemical staining demonstrated positive results for ALKA4, CD68, and CD163 in histiocytes. Weakly positive results were obtained for S-100 and CD45-LCA. Ki-67 labeling index ranged from 3% to 5%. Negative staining results were observed for CD1α, CD21, CD23, CD35, Langerin, Braf (V600E), SSTR2, and CD30. Considering the high surgical risk associated with cauda equina nerve lesion and the absence of evident functional impairment caused by the skin lesion, both of which were small lesions, no further surgical intervention was performed in this pediatric patient to avoid potential iatrogenic injuries.

Postoperatively, a marrow biopsy was performed to exclude other hematological diseases that may coexist. The histological examination showed hyperplasia of the erythroid lineage and prominent presence of megakaryocytes. Cytology result revealed active proliferation of the granulopoietic lineage, relative proliferation of the erythroid lineage, and decreased proportion of lymphocytes, with juvenile lymphocytes accounting for 1.6% of the total count. The marrow flow immunophenotyping result did not show any significant abnormalities. CSF flow immunophenotyping result indicated that 14.2% of cells were suspected to be myeloid naïve-biased stage cells. Postoperative PET/CT imaging did not reveal abnormal metabolism elsewhere. After three courses of chemotherapy with vindesine, CSF flow MRD (minimal residual disease) test revealed no obvious abnormal population of myeloid-derived cells. Following four cycles of chemotherapy and a subsequent 20-day treatment with ALK inhibitor (crizotinib), the size of the lesion in the leptomeninges and the skin became smaller on MR images (**Figures 1L, M**). The patient was ultimately diagnosed with APH, which involved both the nervous system and skin. Subsequently, the patient was instructed to continue oral treatment with crizotinib and other medications outside the hospital. Currently, the patient has shown significant improvement in leg movement.

### 2.2 Literature review

Relevant literature was extensively searched in the PubMed and WanFang databases from 2008 to 2023, without any restrictions on language or study type. The keywords “ALK-positive histiocytosis,” “ALK-rearranged histiocytosis,” and “ALK and histiocytosis” were used for the search. The most recent literature search was conducted in September 2023. Queries were performed across all fields of the papers, including “Case of the Month” section affiliated with the *American Journal of Neuroradiology*. Records were manually collected and deduplicated. The obtained records underwent filtering based on title, abstract, and paper availability. Additionally, we examined bibliography within the literature review to include original reports of all cases. Finally, data were extracted and incorporated into a dedicated spreadsheet.

### 2.3 Data extraction

According to the literature search methods mentioned above, a total of 76 papers covering the period from 2008 to 2023 were obtained. After excluding 2 duplicate records and 44 indirectly related ones, there were 30 papers directly related to APH. Furthermore, after excluding systematic literature reviews, literature unrelated to the nervous system, and literature lacking neurological imaging, a total of 14 literatures that recorded cases of APH involving the nervous system along with corresponding imaging descriptions were obtained. Finally, after excluding one literature with non-standard imaging descriptions, we obtained 3 papers of case series and 10 papers of case report including one in Chinese language, and enrolled a total of 22 patients. In addition to these sources, one case provided by the *American Journal of Neuroradiology* and another case presented in this manuscript were also included. Overall, a total of 24 patients were included in the analysis. We collected information including gender, age, gene fusion mutation, and distribution, number, and longitudinal diameter of lesions in both the central nervous system (CNS) and peripheral nervous system (PNS). Most importantly, we recorded multimodal imaging findings including CT density and MR signal characteristics on plain scans, degree and pattern of MR enhancement, water molecular diffusion characteristic on DWI, and FDG uptake of the lesions.

## 3 Results

To date, less than 100 cases of APH have been documented. In this study, amalgamated with our current case, a total of 24 patients were included, who had involvement of the nervous system and provided imaging descriptions. The age range was from 0.4 to 51 years old, with a median age of 10 years (**Table 1**). Genetically, apart from one case with unknown gene data reported in the *American Journal of Neuroradiology* and three cases showing ALK-FISH positive (including the current one), the remaining 20 cases exhibited KIF5B-ALK gene fusion mutations. Among these 24 cases of APH, a total number of 38 lesions were identified within the nervous system: 27 lesions located within CNS, 10 within the PNS, and 1 affecting both simultaneously. The CNS lesions primarily occurred in gray matter of the cerebral cortex (11/27 lesions) and the subcortical region of the cerebrum (7/27 lesions), while PNS lesions mostly involved the spinal dura/leptomeningeal or nerve roots.

**Table 1:** Imaging findings of neurological involvement in 24 cases of APH.

Patient no.	Sex	Age (years)	Lesion count	Involved area in the nervous system	Maximum diameter (mm)	Density	Perifocal edema	T2WI	T2FLAIR
1 (8)	M	49	2	Gray matter of the cerebral cortex	/	/	/	/	/
2 (9)	M	0.8	2	Gray matter of the cerebral cortex and subcortical region	53	/	Yes	Iso-hypo-	Hyper-
3 (10)	F	17	1	Gray matter of the cerebral cortex	/	/	/	/	/
4 (11)	F	51	1	Gray matter of the cerebral cortex	17	/	Yes	Iso-hypo-	Iso-
5 (12)	M	0.4	1	Gray matter of the cerebral cortex	/	Slightly high	No	Iso-	Iso-
6 (2)	F	10	2	Gray matter of the cerebral cortex and cerebellar cortex (**Figure 3**)	/	/	/	/	/
7 (2)	F	19	1	Spinal nerve root	/	/	/	/	/
8 (2)	F	2	3	Deep gray matter nucleus, deep white matter and subcortical region (**Figure 3**)	/	/	/	/	/
9 (2)	F	28	1	Leptomeningeal (**Figure 3**)	/	/	/	/	/
10 (13)	M	1.5	3	2 at gray matter of the cerebral cortex and 1 at suprasellar	51	/	No	Iso-	Hyper-
11 (7)	M	3	5	2 at gray matter of the cerebral cortex, 2 at subcortical region, and 1 at deep gray matter nucleus	/	Slightly high	No	Iso-	/
12^a^	F	3	2	Intradural extramedullary region and leptomeningeal (**Figure 1**)	27	/	No	Iso-	/
13 (3)	M	15	1	Endocranium	24	/	/	/	/
14 (4)	F	7	1	Gray matter of the cerebellar cortex	30	/	/	/	/
15 (4)	F	10	1	Subcortical region of the cerebrum	14	/	/	/	/
16 (9)	F	11	1	Subcortical region of the cerebrum	15	/	Yes	/	Iso-hypo-
17 (2)	F	13	1	Subcortical region of the cerebrum (**Figure 3**)	/	/	/	/	/
18 (2)	F	3	1	Cranial nerve (**Figure 3**)	/	/	/	/	/
19 (2)	M	11	1	Cranial nerve (**Figure 3**)	34	/	/	/	/
20 (2)	M	12	1	Intradural extramedullary region (**Figure 3**)	45	/	/	/	/
21 (14)	M	3	3	Intradural extramedullary region, spinal dura and leptomeningeal	/	/	/	/	/
22 (15)	F	0.6	1	Thalamus and third ventricle	47	/	No	/	Iso-hyper-
23 (16)	M	30	1	Endocranium	/	Iso-	No	Iso-hypo-	Iso-
24 (17)	M	1.7	1	Leptomeningeal	/	/	/	Iso-hypo-	Iso-hyper-
Patient No.	Intensity of enhancement		Pattern of enhancement					Restricted diffusivity	FDG metabolism
1	Moderate		The large lesion showing homogeneous enhancement with mildly punctate enhancement and the small one homogeneous enhancement with marked ring enhancement					/	High
2	Marked		The large lesion showing inhomogeneous enhancement with marked ring enhancement and the small one inhomogeneous enhancement with a small cystic component					Yes	/
3	Moderate		Homogeneous enhancement					/	/
4	Moderate		Homogeneous enhancement with markedly punctate enhancement					/	High
5	/		/					/	/
6	One moderate, one marked		Homogeneous enhancement with marked ring enhancement					/	/
7	/		/					/	High
8	Moderate		The largest lesion showing inhomogeneous enhancement, the larger one showing inhomogeneous enhancement with marked ring enhancement, and the smallest one showing homogeneous enhancement					/	/
9	Marked		Homogeneous enhancement					/	/
10	Marked		The largest lesion showing homogeneous enhancement with markedly/mildly punctate enhancement, one ring enhancement and another one showing homogeneous enhancement					Yes	/
11	Moderate		The largest lesion showing inhomogeneous enhancement with marked ring enhancement, and the other four showing homogeneous enhancement					Yes	High
12	Moderate		The one showing homogeneous enhancement with markedly punctate enhancement and marked enhancement at the upper and lower rims, the other showing homogeneous enhancement					/	/
13	Moderate		Homogeneous enhancement					/	/
14	Moderate		Inhomogeneous enhancement with marked edge enhancement					Yes	/
15	Moderate		Homogeneous enhancement with mildly punctate enhancement					/	/
16	Mild		Homogeneous enhancement with markedly punctate enhancement and ring enhancement					Yes	/
17	Moderate		Homogeneous enhancement with marked ring enhancement					/	/
18	Moderate		Homogeneous enhancement with marked ring enhancement					/	/
19	Moderate		Homogeneous enhancement with mildly punctate enhancement					/	/
20	Moderate		Homogeneous enhancement with mildly punctate enhancement					/	/
21	Moderate		Homogeneous enhancement					/	/
22	/		/					Yes	High
23	Moderate		Homogeneous enhancement with markedly punctate enhancement and ring enhancement					No	High
24	Marked		Homogeneous enhancement					Yes	/

^a^The case reported here; Patients 1-12 with multisystem involvement, while patients 13-24 only with nervous system involvement.

**Figure 3:**
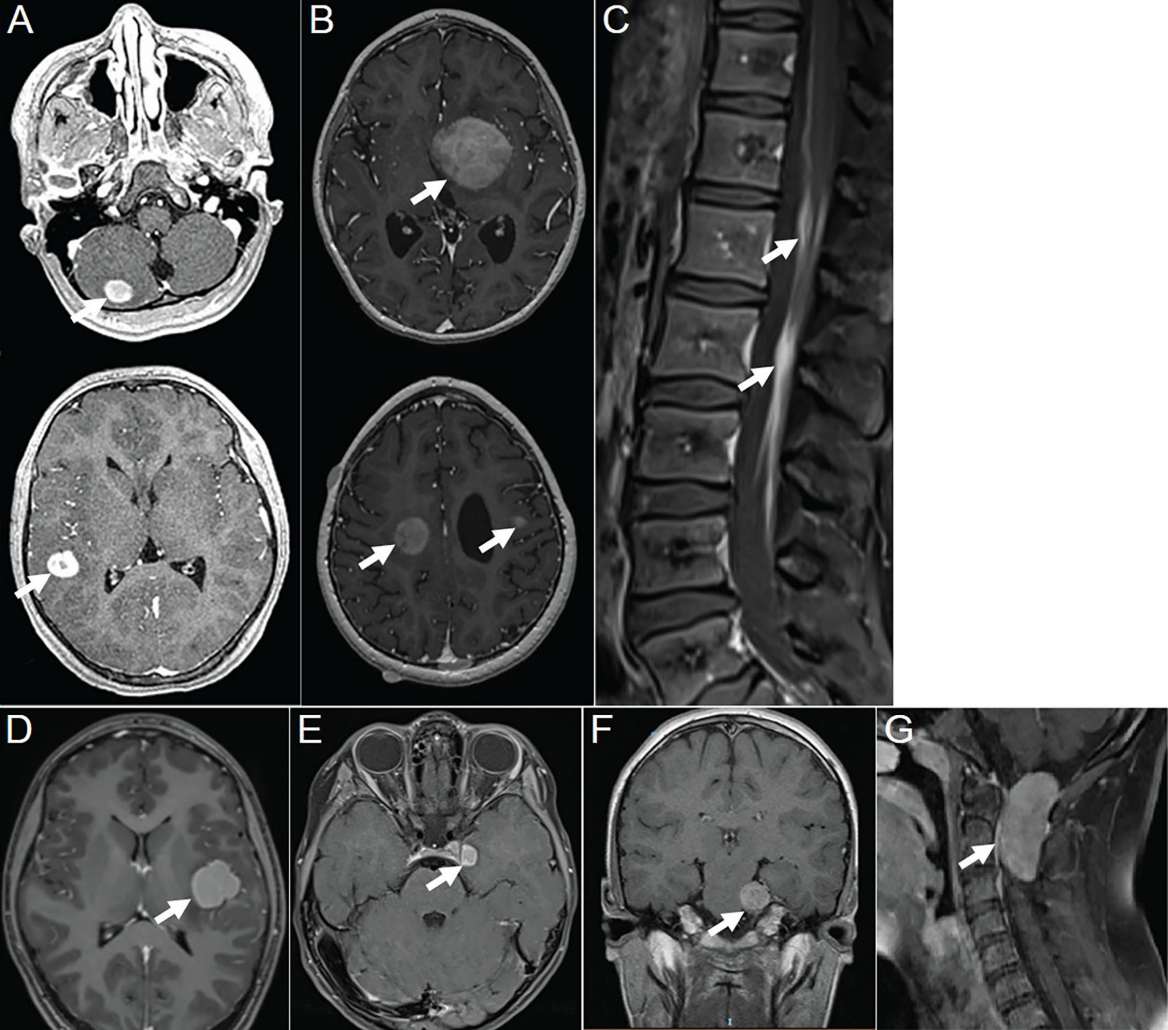
Contrast-enhanced T1WI showing neurologic involvement (arrow) in APH patients. **(A)** Axial images of patient 6 showing a nodule in the gray matter of the cerebellar hemisphere with moderately homogeneous enhancement in the center and marked ring enhancement at the rim, as well as a markedly homogeneously ring-enhanced one in the gray matter of temporal lobe. **(B)** Axial images of patient 8 showing a moderately heterogeneously enhanced lesion in the left basal ganglia, one in the right centrum semiovale with moderately heterogeneous enhancement in the center and marked ring enhancement at the rim, and another one in subcortical region of the left frontal lobe with moderately homogeneous enhancement. **(C)** Sagittal image of patient 9 showing marked and homogeneous enhancement of leptomeningeal along the descending cauda equina nerve roots. **(D)** Axial images of patient 17 showing a lesion located in subcortical region of left insula, with moderately homogeneous enhancement in the center and marked ring enhancement at the rim. **(E)** Axial images of patient 18 showing a tumor located at left oculomotor nerve, with moderately homogeneous enhancement in the center and marked ring enhancement at the rim. **(F)** Coronal image of patient 19 showing a lesion that followed the course of the trigeminal nerve, with moderately homogeneous and mildly punctate enhancement causing pressure on the pons. **(G)** Sagittal image of patient 20 showing a tumor located intradural extramedullary at level C1–C2 with moderately homogeneous and mildly punctate enhancement. Reproduced from (2). Copyright 2022, Elsevier Inc.

The majority of the lesions presented as iso-dense or slightly hyperdense nodules/masses with well-defined and smooth margins, or as thickened cerebral/spinal membranes. Rare cystic component and no indications of calcification or hemorrhage were observed. MR images revealed isointense or slightly hypointense signals on T1WI, isointense, or iso-hypointense signals on T2WI (16/16 lesions), and isointense (6/11 lesions) or hyperintense (5/11 lesions) signals on T2 fluid-attenuated inversion recovery (FLAIR). Apart from a few lesions accompanied by perifocal edema (12/16 lesions), the majority exhibited no signs of mass effect (12/16 lesions). Moderate homogeneous enhancement was predominantly observed on contrast-enhanced T1WI, accompanied by mildly/markedly punctate enhancement or/and smooth ring enhancement at the tumor periphery (**Figure 3**). Most lesions showed varying degrees of diffusion limitation (14/15 lesions), and all exhibited high FDG uptake (11/11 lesions) (**Tables 1**, **2**). Among the 38 lesions, only 3 were located in the intradural extramedullary region and all showed moderate homogeneous enhancement. The two larger lesions were aligned parallel to the spinal canal, and the punctate enhancement in the center was identified within each lesion under magnification (**Figure 3G**). In our case, there was T2 hypointensity observed at the markedly punctate enhancement area, as well as markedly “cap-like” enhancement on both upper and lower rims of the lesion.

**Table 2:** Summary of imaging characteristics in 38 lesions involving the nervous system in APH.

	CT		MRI			Perifocal edema		Intensity of enhancement			Pattern of enhancement			Restricted diffusivity		FDG metabolism
Imaging findings	Iso-dense	Slightly hyperdense	T2WI iso-/iso-hypointense	T2FLAIR isointense	T2FLAIR hyperintense	Yes	No	Mild	Moderate	Marked	Homogeneous	Inhomogeneous	Ring enhancement	No	Yes	High
Lesion ratio	1/7	6/7	16/16	6/11	5/11	4/16	12/16	1/35	26/35	8/35	28/35	6/35	1/35	1/15	14/15	11/11

Additionally, through literature search and statistics, a total of 18 cases (including the present one) of APH patients with skin involvement were identified. The age range varied from neonatal to 71 years old, with a median age of 22.5 months. Infants and young children showed a higher prevalence. Among these cases, 5 were limited to the skin while the remaining 13 cases exhibited concurrent lesions in other organs or systems. These lesions were scattered across the skin of the head, neck, trunk, and limbs without any discernible distribution pattern. The lesions mostly present as maculopapular or nodular with a slightly hard texture and exhibit a dark red or brown color. Genetic analysis revealed that among these cases, 5 showed ALK-FISH positivity; 1 had TFG-ALK fusion; 1 had TPM3-ALK fusion; 1 had COL1A2-ALK fusion; and the remaining 10 all revealed KIF5B-ALK fusion.

## 4 Discussion

We reported a 3-year-old female patient who belonged to the vulnerable population for APH. Based on our literature review, cutaneous involvement appears to be more prevalent in infants and young children with APH compared to neurological involvement, and it seems to involve a greater variety of gene mutations. The etiology of the disease remains unclear. The clinical presentation of the disease is nonspecific, whereas the ambulation difficulty in this case was attributed to spinal cord compression. The diagnosis relies on histopathology and molecular biological detection techniques. ALK inhibitors have shown significant therapeutic efficacy against this disease, as illustrated in this case. The prognosis for the disease is favorable.

The multimodal imaging findings of APH exhibited distinct characteristics: iso-dense or slightly hyperdense on CT, isointense or slightly hypointense on T1WI, iso-hypointense on T2WI, isointense or hyperintense on T2FLAIR, as well as moderate enhancement accompanied by punctate enhancement and/or ring enhancement on contrast-enhanced T1WI. The features observed in CT and MR images indicated dense arrangement of tumor cell or the presence of fibrous component (7). The majority exhibited no mass effect, which could potentially be associated with slow growth of tumors. Smooth ring enhancement at the tumor periphery probably represented chronic inflammatory reaction or the presence of a tumor capsule. The signal characteristics of the lesion on MR images in the present case were similar to those observed in other areas, and the presence of marked “cap-like” enhancement on both upper and lower rims also resembled the characteristic “ring enhancement” sign observed in literatures. Two of three cases located in the intradural extramedullary region revealed a centrally punctate enhancement within the lesion under magnification, resembling mildly/markedly punctate enhancement as presented in other cases. Nevertheless, unlike the relative T2FLAIR hyperintensity observed at the punctate enhancement areas in literature (9, 16), this case exhibited T2-hypointense at such punctate enhancement area. Therefore, further investigation is needed to explore their relationship. Moreover, additional research is required to determine whether these different imaging manifestations in a few cases indicate atypical APH. Additionally, multimodality imaging is also frequently employed for assessing treatment response and prognosis of this disease (2, 13, 15).

APH with neurological involvement is primarily differentiated with other histiocytic tumors such as juvenile xanthogranuloma, Rosai–Dorfman disease, Erdheim–Chester disease, and Langerhans cell histiocytosis (6). The intracranial lesions of juvenile xanthogranuloma predominantly manifest as nodules or masses with iso-height signal on T1WI, located adjacent to the ventricles or meninges and involving the brain parenchyma (18, 19), while intracranial APH is more likely to be observed within the gray matter of the cerebral cortex and subcortical region of the cerebrum. The intracranial manifestation of Rosai–Dorfman disease on MR is similar to that of APH, but the former may exhibit marginal “burr sign” and “meningeal tail sign” resembling those seen in meningioma (20, 21). Both Erdheim–Chester disease and Langerhans cell histiocytosis belong to Group L histiocytosis, with bone involvement being the most common manifestation (22). Intracranially, ECD typically affects the posterior fossa. The predominant imaging findings include multifocal FLAIR hyperintensities and variable enhancement involving the dentate nuclei and brainstem (20). By contrast, Langerhans cell histiocytosis is characterized by nodule within the hypothalamic–pituitary axis and loss of the hyperintensity in posterior pituitary lobe, which are also occasionally observed in Erdheim–Chester disease (22). Additionally, when encountering solitary focal APH located at the endocranium, differentiation from meningioma should also be considered, whereas in the present case, the lesion was located in the intradural extramedullary region and should be differentiated from neurogenic tumor.

## 5 Limitation

The existing literature on APH primarily consists of case reports that utilize various imaging equipment with inconsistent parameters to obtain incomplete or inconsistent images, thereby limiting the applicability of the data.

## 6 Conclusion

APH is an extremely rare neoplasm of histiocytes that predominantly involves the nervous system, particularly the gray matter of the cortex and subcortical region of the cerebrum in CNS. The majority of the lesions typically present as CT iso-dense or slightly hyperdense, T1WI isointense or slightly hypointense, T2WI iso-hypointense, and T2FLAIR isointense or hyperintense. They exhibit moderate enhancement accompanied by punctate enhancement and/or ring enhancement on contrast-enhanced T1WI. Furthermore, high DWI signal and FDG uptake are also characteristic features of this disease. Familiarity with these imaging features will improve preoperative diagnostic accuracy for this disease.

## Data availability statement

The original contributions presented in the study are included in the article/supplementary material. Further inquiries can be directed to the corresponding authors.

## Ethics statement

The studies involving human participants were reviewed and approved by the Medical Ethics Committee of Tongji Hospital affiliated to Tongji Medical College, Huazhong University of Science and Technology. Written informed consent was obtained from the minor’s legal guardian for the publication of any potentially identifiable images or data included in this article. For all the cases in **Table 1**, written informed consents were obtained from the individuals or the minors’ legal guardians for the publications of any potentially identifiable images or data included, when the literatures were first published.

## Author contributions

JW: Writing – original draft, Writing – review & editing. YZ: Investigation, Writing – original draft. YX: Formal analysis, Methodology, Writing – review & editing.
